# COVID-19 and cancer research

**DOI:** 10.1038/s41416-020-0960-1

**Published:** 2020-06-26

**Authors:** Adrian L. Harris

**Affiliations:** Editor-in-Chief, BJC Editorial Office, British Journal of Cancer, Cancer Research UK, 2 Redman Place London, London, E20 1JQ UK

## Abstract

The COVID-19 pandemic has had a devastating effect on human lives and society. The accompanying editorial summarises some of the major effects on cancer patients and impacts on cancer research. These may be mitigated by appropriate responses from governments, research funders, charities, universities, industry and the public. It is already clear that different approaches to management have drastically different outcomes.

## Main

COVID-19 has had a major impact on everything we do, but in this editorial, I will consider the cancer field. Cancer patients are at the forefront of risk from COVID-19 and have a disproportionately high death rate. Clearly, some of these factors relate to immune suppression and chemotherapy, which means the relative risks of treatment versus death from coronavirus versus infection need to be carefully balanced. Also, the need for isolation of patients and delivery of treatment is a problem, particularly intravenous and intramuscular injections. This has led to prioritisation lists, ranking from the most curable, to those least likely to benefit. No trial therapies can be used, as all trials are stopped for cancer therapy. Highly innovative clinical trials of advanced cellular therapies, such as tumour-infiltrating lymphocytes (TILs) and chimaeric antigen receptor (CAR)-T cells, will be particularly adversely affected as these also often require input from critical care/intensive therapy unit (ITU). There will also be consequences on survival for those patients treated with curative intent for whom critical care is often required to deliver their treatment (e.g., major thoracic surgery or oesophagectomy and bone marrow transplantation in haemato-oncology), and which has been delayed due to de-prioritisation as ITU has been focused on the COVID-19 pandemic.

With a lead time of 4–5 weeks to learn and plan, we know full personal protective equipment (PPE) use in China, Singapore and South Korea to be necessary for staff protection, and there were no deaths of Chinese doctors once this was followed. Already, dozens of medical staff have died in Italy and in many other countries (Iran, Egypt, United Kingdom and United States, see *in memoriam*  at https://www.medscape.com/viewarticle/927976?nlid=134809_3903&src=wnl_exclsv_200402_MSCPEDIT&uac=307247DX&impID=2332449&faf=1).

The effects of the virus on drug production and the pharmaceutical industry have not dented supplies yet, and there has been a strong response from the industry. Indeed, many companies offered major facilities to rapidly extend testing, as, for example, in Germany, which is doing the most tests per head of population in Europe.

The long-term impact on therapy is hard to evaluate, but many trials are on hold, from practice-changing to novel agents. New drugs will not be offered to patients for many months to come. The research effort across the community, academics, government and industry, is halted, and many complex models of cancer had to be shut down. The economic consequences may also irreversibly damage the biotechnology industry, which is often at the forefront of innovative diagnostics, and the development of novel therapies.

The careers of many will be affected, including undergraduate students, PhD students, technicians, postdoctoral scientists and the key staff that maintain laboratories. This interruption would prevent them finishing their degrees or PhDs on time; their promotion and principal investigators will not be able to apply for or initiate grants. It is critical that the funding agencies work together to solve this problem, including medical research councils, charities, government and academic institutes. A unified proposal is necessary to allow extension of grants and salaries over the period of shutdown, considering that there will be no expense on consumables.

Much of the research in cancer is funded by charities or government funds, both of which will be severely depleted. All charities will be severely hit because of loss of income from public donors, grants and industry. Most charities run on 6-month reserves to keep their work at full pace without any income. Governments’ response to these challenges will change the future face of scientific research and charitable work. Charities can continue to help with curated advice to their supporters.

The universities, which are such a source of innovation and development and the main site of research cancer, are under severe threat because of loss of funding, particularly from overseas students. There are many willing investigators and postdoctoral fellows in these centres with access to PCR machines and full laboratory facilities that would have been willing to expand virus testing, but in many countries, they have not been recruited.

Overall, the response of the populations is highly impressive, particularly in the volunteering and preparedness of staff essential for continued supplies of all types, energy, water, food, fuel and physical communication systems, and electronic communications, e.g., banks, telephones and the Internet. The medical and allied professions have been outstanding, at great personal risk in many countries because of lack of PPE.

But it is disappointing that the science has not been used to its full extent, since we already knew of problems in China, the Gates Foundation had modelled this exact scenario and previous planning exercises in 2016 had worked out the exact resources we now need. The basic principles of handling epidemics were ignored by many governments not using Chinese data, the proper use of PPE for staff, masks for the population and the protection of the general population by contact tracing and general testing. Recent advances in AI to manage this could be helpful (https://www.linkedin.com/pulse/partnering-nhs-digital-public-health-england-mihaela-van-der-schaar).

These should have been initiated in January. It will be important to see how the death rates vary between a country like the United States, which was so late and others, like Singapore, South Korea and Taiwan, which were early in isolation, testing and protecting their populations. This may help understanding herd immunity and dealing with expected further rounds of infection. The overall poor response was based on lack of disaster planning and choosing not to follow the science and empirical data already present. Planning for previous influenza epidemics in the United Kingdom, including fitting all general practitioners with personal protective masks, was well organised, but turned out to be unnecessary.

There are many guidelines becoming available for patients with different types of cancer, so we may soon need guidelines to the guidelines. Many major general medical and specialist journals are providing needed advice. Reliable and honest communication is essential, and unfortunately, we have already seen the problems caused by the initial Chinese suppression of information, the USA response of ignoring the threat or ‘COVID denial’, and advice continually fluctuating in the United Kingdom on testing the population or not, testing staff, masks or not and full PPE for medical staff or not (not depending on science but on availability of PPE).

The *British Journal of Cancer* will continue publishing and welcomes comments, articles, reviews and correspondence on COVID-19 virus and its effects on cancer researchers and patients.

## Data Availability

Not applicable

